# The association between screen time exposure and myopia in children and adolescents: a meta-analysis

**DOI:** 10.1186/s12889-024-19113-5

**Published:** 2024-06-18

**Authors:** Zhiqiang Zong, Yaxin Zhang, Jianchao Qiao, Yuan Tian, Shaojun Xu

**Affiliations:** 1 The Second School of Clinical Medicine, Anhui Medical University, 81 Meishan Road, Hefei, Anhui 230032 China; 2 Department of Maternal, Child and Adolescent Health, School of Public Health, Anhui Medical University, 81 Meishan Road, Hefei, Anhui 230032 China; 3 The First School of Clinical Medicine, Anhui Medical University, 81 Meishan Road, Hefei, Anhui 230032 China; 4 MOE Key Laboratory of Population Health Across Life Cycle, 81 Meishan Road, Hefei, Anhui 230032 China

**Keywords:** Myopia, Children, Screen time, Meta-analysis, Public health

## Abstract

**Objective:**

This study aimed to systematically review epidemiological evidence on associations between screen time exposure and myopia in children and adolescents, and to quantitatively evaluate summary effect estimates from existing literature.

**Method:**

There were three online databases including PubMed, Embase, and Web of Science, for epidemiological studies on screen time exposure and myopia published before June 1, 2023. The risk of bias was assessed by the Newcastle Ottawa Scale (NOS) checklist. Summary odds ratios (ORs) and 95% confidence intervals (CIs) were calculated to evaluate the correlation between screen time exposure and myopia using random or fixed-effect models by exposure type (categorical/continuous). We also performed subgroup analysis by screen device type, study quality, geographic region, and research period.

**Results:**

We searched 7,571 records from three databases and identified 19 eligible studies, including 14 high-quality studies and 5 moderate-quality studies. Meta-analyses suggested that there was a statistically significant correlation between screen time (high vs. low) and myopia. The pooled ORs with 95%CIs were respectively 2.24 (1.47–3.42) for cross-sectional studies, and 2.39 (2.07–2.76) for cohort studies. We also found a significant association between continuous exposure to screen time (per 1 h/d increase) and myopia in cohort studies. The pooled ORs with 95%CIs were 1.07 (1.01–1.13). In subgroup analysis stratified by screen device type in cross-sectional studies, screen time exposures from computers (categorical: OR = 8.19, 95%CI: 4.78–14.04; continuous: OR = 1.22, 95%CI: 1.10–1.35) and televisions (categorical: OR = 1.46, 95%CI: 1.02–2.10) were associated with myopia, while smartphones were not. Although publication bias was detected, the pooled results did not show significant changes after adjustment using the trim and fill method.

**Conclusion:**

Our findings support that screen time exposure was significantly associated with myopia in children and adolescents. Notably, screen time exposure from computers may have the most significant impact on myopia.

**Supplementary Information:**

The online version contains supplementary material available at 10.1186/s12889-024-19113-5.

## Introduction

Myopia, also known as near-sightedness or short-sightedness, is one of the refractive errors [1]. The feature of myopia is the excessive elongation of the ocular globe [2]. The risk of pathological eye changes like cataracts, glaucoma, retinal detachment, and macular degeneration can be increased by high myopia, all of which can lead to irreversible vision loss. Consequently, myopia brings further vision challenges [3–6]. It is evident that environmental factors, such as education, near-work activities, and outdoor activities exert a significant influence on the prevalence of myopia [7–9]. In order to effectively prevent and control myopia, it is necessary to explore more potential factors that may affect myopia.

Screen time includes computers, televisions, video games, and other mobile digital devices (e.g., smartphones, laptops, and tablets) [10–12]. Screen time exposure has become a ubiquitous part of children’s and adolescents’ day-to-day lives [13]. In recent years, with the popularity of screen devices, children have more chances to be exposed to screen every day, while the age of first exposure to screen time has been reduced [14]. At the same time, the available screen devices and their range of use have rapidly expanded. Television used to be the primary mode of viewing screens in homes, but modern screen devices that children can use include mobile digital devices, such as computers, smartphones, and tablets [15]. Through e-books, games, and custom applications, children can gain more opportunities for cognitive engagement. When combined with the interactive nature of these activities with high-quality educational content, children may derive benefits, including improved language and motor skills, and strengthened social connections [16–18]. However, there is sufficient evidence to demonstrate the relationship between excessive screen time and negative health outcomes in children and adolescents, such as hypertension [19], insulin resistance [20], overweight/obesity [21], sleep disorder [10] and depression [22]. In 2018, the Chinese Ministry of Education imposed controls on the use of electronic products, stating that “the single use of electronic products for non-learning purposes should not exceed fifteen minutes, and the cumulative daily use should not exceed one hour” [23]. The World Health Organization (WHO) has proposed a recommendation to limit screen time to no more than two hours per day in 2020 [24]. In this field, the extent to which modern screen time exposure is beneficial or harmful to the health and development of children and adolescents remains an active research topic.

In recent years, an increasing number of epidemiological studies have estimated screen time exposure through self-reported questionnaires. Several studies have shown a positive correlation between screen time exposure and myopia [25–27], while others have reported null results [28–30]. Given that screen time exposure is almost ubiquitous in modern social life, researchers further elucidate that the relationship between screen time exposure and myopia is a major public health issue. As far as we know, previous systematic reviews have reported that the results of screen time and myopia were mixed [9, 31]. In general, the findings of published observational studies in relevant fields are contradictory, and the literature reviewed and included in relevant reviews need to be further supplemented and improved.

To fill these gaps, we conducted a systematic review and meta-analysis based observational studies that assessed the relationship between screen time exposure and myopia in children and adolescents. Screen time exposure types include categorical exposure and continuous exposure, and screen device types include computers, televisions, smartphones, laptops and tablets, etc. When data were available, we performed quantitative synthesis to calculate pooled effect estimates. We assessed the risk of bias in individual studies and rated the quality of evidence across studies.

## Materials and methods

When conducting this systematic review and meta-analysis, two authors (ZZ and YZ) conducted literature screening, data extraction, risk of bias assessment, evidence synthesis and analysis. A third author (SX) was necessary if there was any disagreement between the two authors. The Preferred Reporting Items for Systematic Reviews and Meta-Analysis (PRISMA) 2020 statement was followed to perform this meta-analysis (Table S1) [32].

### Eligibility criteria

According to the PECOS statement, the eligibility criteria for Population, Exposure, Comparator, Outcomes, and Study Design are as follows: (P) studies of children and adolescents; (E) studies on categorical or continuous screen time exposure; (C) studies presenting comparative effect estimates (i.e., compared with children exposed to different levels of screen time) or continuous effect estimates (i.e., the impact on myopia by one hour of screen time increase per day); (O) Studies reporting the incidence or prevalence of myopia in children and adolescents. Adjusted odds ratios (ORs) and 95% confidence intervals (CIs) for myopia must be used; (S) Study design–published observational studies using cross-sectional, cohort, or case-control study designs. If more than one publication for the same research population was found, the most recent one with the most comprehensive and informative was selected [33].

### Search strategy and study selection

We searched PubMed, Embase, and Web of Science for related literature from inception to 1st June, 2023.

The search terms are as follows: (screen OR “digital screen” OR television OR computer OR smartphone OR tablet OR “electronic device”) AND (myopia OR “refractive error” OR “near sightless”) AND (children OR teenagers OR adolescents) (see Table S2 for details). The database search was restricted to studies published in English. Additionally, references and related reviews of identified articles were manually scanned, and a follow-up search was conducted prior to manuscript submission to identify qualified published data.

Studies were excluded from this study if their characteristics were as follows: (I) reviews, letters, or commentaries; (II) occupational exposure studies; (III) non-human studies; (IV) ecological studies; (V) studies without available effect estimates for this meta-analysis. After removing duplicated studies, two authors (ZZ and YZ) screened titles, abstracts, and full text for eligibility using EndNote X9. If there was any discrepancy between the two authors, a third author (SX) was required.

### Data extraction and quality assessment

From each study, two investigators (ZZ and YZ) independently extracted the following information: first author and publication year, country and geographic region, study design, sample size and age, data source, screen device type, definition and examination of myopia, main results (ORs and 95%CIs), and adjustment for confounders. In order to gather unpublished data, a direct contact was made with authors when it was considered appropriate. The most fully adjusted effect estimate was used to perform the meta-analysis, which represents the greatest control over potential confounders. Data on screen time exposure were collected through questionnaires in the included studies.

Newcastle Ottawa Scale (NOS) evaluations, based on nine-star scoring system (one star represents one score), were conducted for cohort, panel, and case-crossover studies. And an adapted form of NOS for cross-sectional studies was also applied in this study [34]. The NOS consists of three categories: outcomes, comparability, and selection. An NOS score of 0 to 10 was used to evaluate studies, with a score greater than or equal to 7 suggesting high quality, a score between 5 and 6 indicating moderate quality, and a score less than or equal to 4 indicating low quality [35]. More detailed criteria for risk of bias assessment can be found in Table S3-S4.

### Statistical analysis

In this meta-analysis of observational studies, R 4.3.1 software, including “meta” and “metafor” packages was used for statistical analysis. In this study, we applied the high versus low category method for studies with categorical exposures after a preliminary assessment of included studies [36]. For continuous exposure, we also extracted and standardized effect estimates for each 1 h/d increase in screen time exposure. We calculated log OR and standard error (SE) using OR and 95% CI that had been reported for the relationship between screen time exposure and the myopia in children and teenagers. Then, heterogeneity analysis was performed using Cochrane Q’ test and inconsistency index (I^2^) [37]. The fixed-effect model for analysis is used if *P* ≥ 0.05 and I^2^ ≤ 50%, while I^2^ ≤ 25% indicates low heterogeneity and 25–50% indicates moderate heterogeneity among included studies; the random-effect model for analysis is used if *P* < 0.05 and I^2^ > 50%, which indicate high heterogeneity among studies [38]. We performed subgroup analysis stratified by screen device type, study quality, geographic region, and research period. We divided the research period into before and after 2008, because the widespread use of smart devices can be traced back to after 2008, especially with the development of smartphones [39]. In our study, sensitivity analysis using the leave-one-out method was applied for the purpose of testing the meta-analysis results’ robustness. We applied funnel plots, and Egger’s test, to evaluate the publication bias of the outcome [40]. The presence of publication bias is suggested if the funnel plot is significantly asymmetric by visual inspection, as well as the *P* value of Egger’s test is greater than 0.05. If publication bias is observed, the trim and fill method will be applied to correct the pooled results [41].

## Results

### Characteristics of included studies

**Fig. 1:**
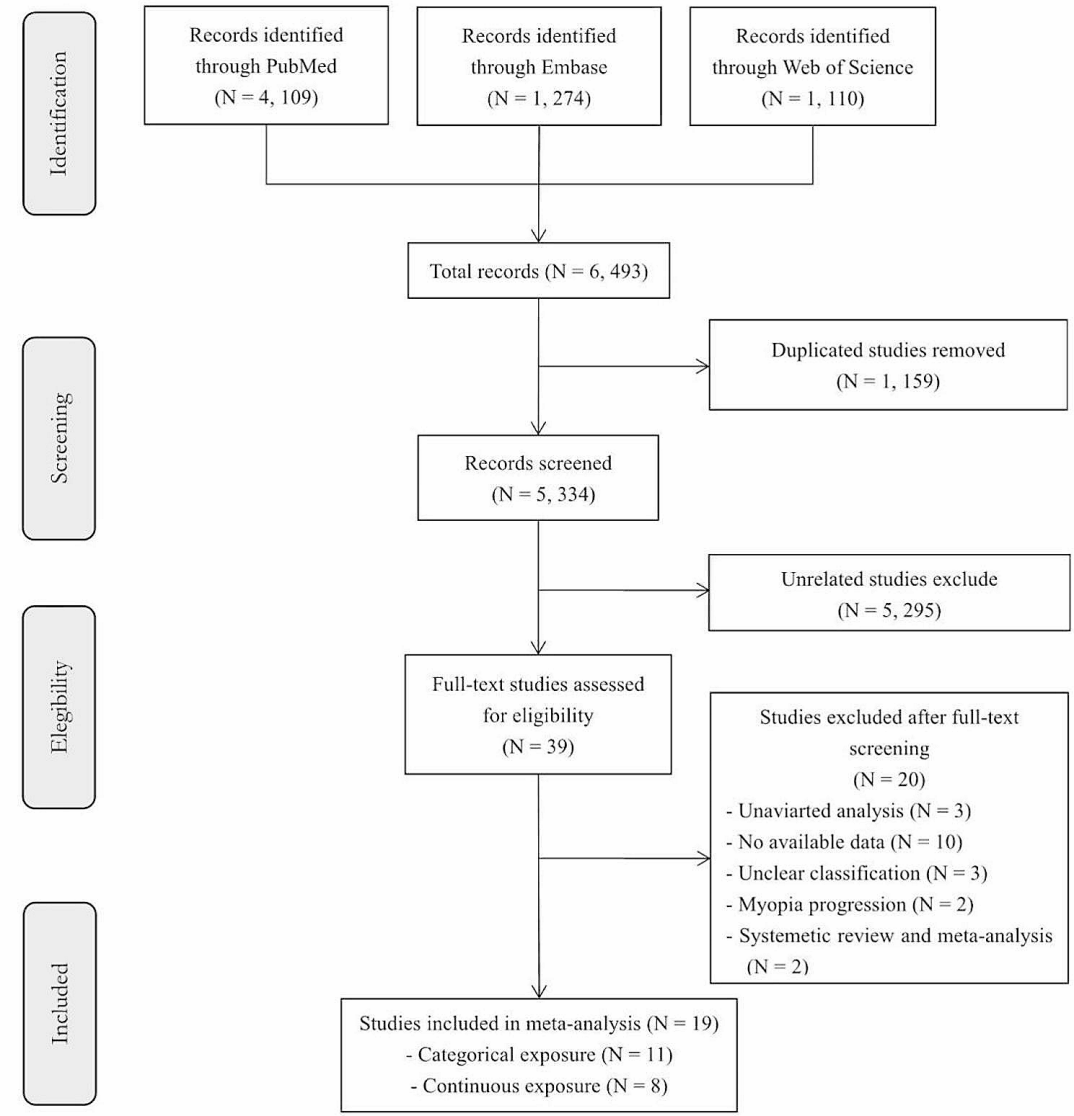
The flowchart of literature search

The flow chart of the literature search was presented in Fig. 1. A total of 6,493 articles were identified from PubMed, Embase, and Web of Science. 1,159 duplicated studies were excluded. After deleting 5,295 unrelated studies during the screening by the titles and abstracts, 39 articles were assessed for eligibility. After scanning the full text, 20 articles were excluded due to univariate analysis, no available data, no myopia prevalence, etc. (see Table S5 for details of exclusion). Finally, we included 19 eligible studies.

In total, 102,360 participants were involved in all included studies, 91,282 in cross-sectional studies (*N* = 15) and 11,078 in cohort studies (*N* = 4). Meanwhile, 13 (68%) studies used cycloplegic refraction, three studies (16%) used self-reported myopia [28, 51, 52], and three studies (16%) performed optometry with a noncycloplegic state [27, 53, 54]. The included studies were from nine countries, two studies (10%) were conducted in North America [27, 54], seven (38%) in Europe [25, 28–30, 45, 46, 52], six (32%) in East Asia [43, 44, 47, 48, 50, 53], two (10%) in South Asia [26, 49], and two (10%) in Southeast Asia [42, 51] (Table 1).

**Table 1:** Characteristics of included studies

First author and Publication year	Country and geographical region	Study design	Sample size and age	Data source and research period	Exposure assessment	Definition and examination of myopia	Screen device type and Main results (OR and 95%CI)	Adjustment for confounders
Berticat et al., 2020	France, Europe	Cross-sectional	264, 4–18 years old	Children’s visit data at Montpellier University Hospital Center from May 2017 to May 2018	Questionnaire	Myopia was defined as SE < 0.50 D. All included children underwent a complete ophthalmologic examination, including refraction under cycloplegia, slit-lamp examination, and dilated fundus examination.	Screen-based electronic devices tablets, cell phones, video games and computers, per 1 h/d increase: OR = 2.33 (0.89–6.05)	Age, sex, BMI, reading time, outdoor time, mother myopia, father myopia, sport, refined carbohydrates consumption.
Chiang et al., 2019	USA, North America	Cross-sectional	9960, 12–19 years old	United States National Health and Nutrition Examination Survey (NHANES) 1999–2008 dataset.	Questionnaire	Myopia was defined as SE ≤ -1.00 D in the worst eye. Refractometry was performed in all children in a non-cycloplegic state by autorefractometry.	Television, ≤ 1 h/d: reference 2 h/d: OR = 1.31 (1.07–1.61) ≥ 3 h/d: OR = 1.49 (1.18–1.87)	Gender, household smoker, education (grade), family poverty income ratio, hours of computer use, serum vitamin D, sugar intake, race.
Chua et al., 2015	Singapore, Southeast Asia	Cohort	572, 30–40 months	Data from the Growing Up in Singapore Towards Healthy Outcomes (GUSTO) birth cohort (naturally conceived) and the In Vitro Fertilization (IVF) cohort consist of offspring of pregnant women aged 18 years and above during June 2009 and September 2010.	Questionnaire	Myopia was defined as SE ≤ -0.50 D. Cycloplegic refraction was performed using an auto-refractor.	Computer, per 1 h/d increase: OR = 0.92 (0.31–2.74) Tablet, per 1 h/d increase: OR = 1.04 (0.67–1.61)	Age, sex, ethnicity, maternal education level, parental myopia, and height at 36 months.
Deng et al., 2010	USA, North America	Cohort	147, 6–8 years old	Data from a longitudinal study of refractive error and visual function that started in 1974 at MIT and continued at NECO until 2006	Questionnaire	Myopia was defined as SE < 0.50 D. Non-cycloplegic distance retinoscopy was used to refract the children, performed by one experienced optometrist	Computer, per 1 h/d increase: OR = 1.03 (0.95–1.12) Television, per 1 h/d increase: OR = 1.07 (1.01–1.13)	Age, myopic parents
Guan et al., 2019	China, East Asia	Cross-sectional	19,934, 9–12 years old	Data from a survey on 252 primary schools in Northwest China, in the fall of 2012.	Questionnaire	Myopia was defined as VA ≤ 6/12 and SE≤ -0.5D in at least one eye. If a child’s VA was 6/12 in either eye, cycloplegia was applied with up to 3 drops of cyclopentolate 1%, and a refractionist, previously trained by experienced pediatric optometrists, carried out automated refraction with subjective refinement in each eye separately.	Smartphone, 0 h/d: reference 0–0.5 h/d: OR = 1.03 (0.94–1.12) 0.5–1 h/d: OR = 0.99 (0.81–1.20) > 1 h/d: OR = 1.17 (0.93–1.48)	Grade, age, sex, family wealth, both parents out-migrated for work, maternal education, child’s main residence, after-school study time, before-school outdoor time, mid-day outdoor time, after-school outdoor time.
Guo et al., 2016	China, East Asia	Cross-sectional	3055, 6–15 years old	Data was from a survey of primary and middle school students attending public or private schools in December 2014, in Guangzhou.	Questionnaire	Myopia was defined as SE ≤ -0.50 D, cycloplegic auto refraction, and subjective refinement were performed by an ophthalmologist in each eye.	Television, 0 h/w: reference 0–2 h/w: OR = 1.54 (1.16–2.05) 2–4 h/w: OR = 1.60 (1.22–2.08) > 4 h/w: OR = 1.96 (1.38–2.77)	Gender, grade, time of reading for pleasure, reading distance, distance of watching TV, myopic parents.
Hagen et al., 2018	Norway, Europe	Cross-sectional	393, 16–19 years old	Data from a survey on two upper secondary schools in Southeast Norway during 2015–2016.	Questionnaire	Myopia was defined as SE ≤ -0.50 D. Cycloplegic refraction was performed using an auto-refractor.	Near electronic devices (smartphones, tablets, and computers), per 1 h/d increase: OR = 1.01 (0.78–1.31)	Sex, age, reading paper, outdoor activities, indoor activities.
Hansen et al., 2019	Denmark, Europe	Cohort	1443, 16–17 years old	Data was from the Copenhagen Child Cohort 2000 (CCC2000), which is a prospective, population-based, observational study initiated in the year 2000.	Questionnaire	Myopia was defined as SE (spherical equivalent) ≤ -0.50 D. The participants were asked to read as many letters as possible with their autorefractor correction. Then + 0.50 D lenses were added consecutively until a significant blur occurred. Hereafter, 0.25 lenses were added until maximum visual acuity was achieved and denoted as the best-corrected visual acuity (BCVA).	Screen-based electronic devices including smartphones, tablet and computers, < 2 h/d: reference 2–4 h/d: OR = 1.89 (1.09–3.28) 4–6 h/d: OR = 1.68 (0.98–2.89) > 6 h/d: OR = 1.89 (1.10–3.24)	Age, sex, weight, height, and physical activity.
Harrington et al., 2019	Ireland, Europe	Cross-sectional	1626, 6–13 years old	Data from Ireland Eye Study (IES) in the period of June 2016 to January 2018	Questionnaire	Myopia was defined as SE ≤ -0.50 D. Once cycloplegia had been achieved, at least 20 min after the instillation of the eye drops, autorefraction was carried out.	Smartphone, < 1 h/d: reference 1–3 h/d: OR = 1.67 (1.00-2.67) > 3 h/d: OR = 3.33 (2.00–5.00)	Age, ethnicity, after-school activities, reading/writing in leisure time, daylight exposure during summer, birth season, breastfed, bottle-fed, BMI, parental myopia.
Harrington et al., 2023	Ireland, Europe	Cross-sectional	723, 6–8 years old	Data was from a survey of children in mainstream schools in Ireland. Data collection occurred between June 2016 and January 2018.	Questionnaire	Myopia was defined as SE ≤ -0.50 D. Cycloplegic refraction was performed using an auto-refractor.	Daily screen time including computers, Nintendo games, iPads, smartphones or televisions, 0–2 h/d: reference > 2 h/d: OR = 10.9 (4.4–27.2)	Age, ethnicity.
Liu et al., 2019	China, East Asia	Cross-sectional	566, 6–14 years old	Data from a survey conducted in urban areas of Tianjin, China, was collected from November 2016 to July 2017.	Questionnaire	Myopia was defined as an SER < -0.50 D in the right eye. Cycloplegic refraction was performed using an auto-refractor.	Smartphone, per 1 h/d increase: OR = 1.30 (1.07–1.48)	Age, sex, BMI, monthly family income, parental myopia, time spent outdoors, time spent reading and writing, reading and writing distance, and daily sleep duration.
McMcrann et al., 2020	Ireland, Europe	Cross-sectional	418, 12–20 years old	Data from a survey conducted in Ireland from January to March 2018.	Questionnaire	Self-reported myopia and wearing glasses/contact lenses for myopia.	Smartphone, per 1 min/d increase: OR = 1.03 (1.00-1.05) Smartphone, per 1 h/d increase: OR = 4.66 (1.08–20.13)	Age, gender, number of myopic parents.
Qian et al., 2016	China, East Asia	Cross-sectional	7681, 5–16 years old	Data from a school-based eye survey, which was conducted in Mangshi located in the middle part of Yunnan province in 2014	Questionnaire	Myopia was defined as SE < 0.50 D. Cycloplegic refraction was performed using an auto-refractor.	Computer, per 1 h/d increase: OR = 1.17 (1.03–1.32)	Age, sex, ethnicity, height, time outdoors, time on reading, having myopic father, having myopic mother
Saxena et al., 2015	India, South Asia	Cross-sectional	9884, 9–14 years old	Data from a survey conducted among children studying in classes 1 to 9 in different schools of Delhi for sub-normal vision and prevalence of myopia, 2014.	Questionnaire	Myopia was defined as SE < -0.50 D. Cycloplegic auto refraction and subjective refinement by an ophthalmologist in each eye.	Computer/video games, 0 h/w: reference 1–4 h/w: OR = 4.50 (2.33–8.98) > 4 h/w: OR = 8.10 (4.05–16.3)	Age, gender, school type, myopic parents, time for reading/writing, outdoor activities.
Schuster et al., 2020	Germany, Europe	Cross-sectional	32,663, 0–17 years old	Data from the German Health Interview and Examination Survey for Children and Adolescents (KiGGS) 2003–2006 and 2014–2017	Questionnaire	Self-reported or parents-reported myopia.	Computer, 0 h/d: reference 0–1 h/d: OR = 1.28 (0.88–1.87) 1–2 h/d: OR = 1.40 (0.96–2.06) > 2 h/d: OR = 1.25 (0.86–1.83)	Age, sex, socioeconomic status (based on the reported occupation, education and parental income) and migration background.
Singh et al., 2019	India, South Asia	Cross-sectional	1234, 7–14 years old	Data was from research performed on schoolchildren studying in grades 1 to 10 in two private schools of Gurugram, Haryana, during 2011–2018.	Questionnaire	Myopia was defined as spherical equivalent refraction of − 0.50 D or greater myopia in either or both eyes. Refraction was performed on all the referred children by the same two optometrists in two stages: cycloplegic refraction and subjective acceptance after 1-week visit (post-mydriatic subjective acceptance).	Computer/video game, 0–2 h/d: reference > 2–4 h/d: OR = 8.33 (3.54–19.58) > 4 h/d: NA	Sex, age, family history, spherical equivalent refractive error, outdoor play hours, study hours.
Toh et al., 2019	Singapore, Southeast Asia	Cross-sectional	1884, 10–18 years old	Data from an online survey to adolescents recruited from schools in Singapore during 2017–2018.	Questionnaire	Self-reported myopia	Smartphone, per 1 h/d increase: OR = 0.97 (0.94–0.99) Tablet, per 1 h/d increase: OR = 0.99 (0.94–1.05)	Gender, grade at school, mental health score, amount of physical activity and total duration of technology use.
Tsai et al., 2020	China, East Asia	Cohort	8916, 3–18 years old	Eight face-to-face myopia surveys were conducted in Taiwan between 1983 and 2017.	Questionnaire	Myopia was defined as SE ≤ -0.25 D. Cycloplegic refraction was performed by instilling three successive drops of 0.5% tropicamide at five-minute intervals, carrying out refraction using autorefractometers.	Screen-based electronic devices including smartphone, tablet and desktop, 0–1 h/d: reference > 1 h/d : OR = 2.43 (2.10–2.81)	Age, sex, sleep, outdoor activities, and near-work activities.
Xie et al., 2020	China, East Asia	Cross-sectional	997, 7–13 years old	Data from a cross-sectional survey of the prevalence of myopia and its associated risk factors in children attending primary schools from grades 1 to 6 in Dianjiang County, Chongqing, China, 2018.	Questionnaire	Myopia was defined as SE < -0.50 D, refractometry was performed in all children in a noncycloplegic state by autorefractometry.	Television: 0–1 h/d: reference 1–2 h/d: OR = 1.41 (0.98–2.03) 2–3 h/d: OR = 1.13 (0.68–1.88) ≥ 3 h/d: OR = 2.47 (1.48–4.15)	Age, gender, myopic parents, eye exercises, outdoor activities, homework time.

### Assessment of risk of bias

As presented in Table S6, according to the NOS checklist, 14 studies (74%) with a score ≥ 7 stars were considered high quality [26, 29, 30, 42–51, 53], while the remaining five studies (26%) with 5 or 6 starts were considered moderate quality [25, 27, 28, 52, 54]. The results of bias risk assessment using the NOS checklist revealed the following possible sources of bias: the sample size included in six (32%) studies was relatively small [25, 29, 30, 47, 52, 53]; five studies (26%) had insufficient strategies to deal with confounding factors (e.g. gender or age) [25, 27, 28, 46, 54], while five studies (26%) lacked adjustment for key confounding factors (e.g. outdoor activities) [28, 30, 42, 44, 52]; five studies (26%) did not use cycloplegic refraction to confirm myopia cases [28, 51–54]; four studies (21%) did not explicitly demonstrate in the main text that there were no results of interest at the beginning of the study [42, 45, 50, 54].

### Screen time exposure and myopia

**Fig. 2:**
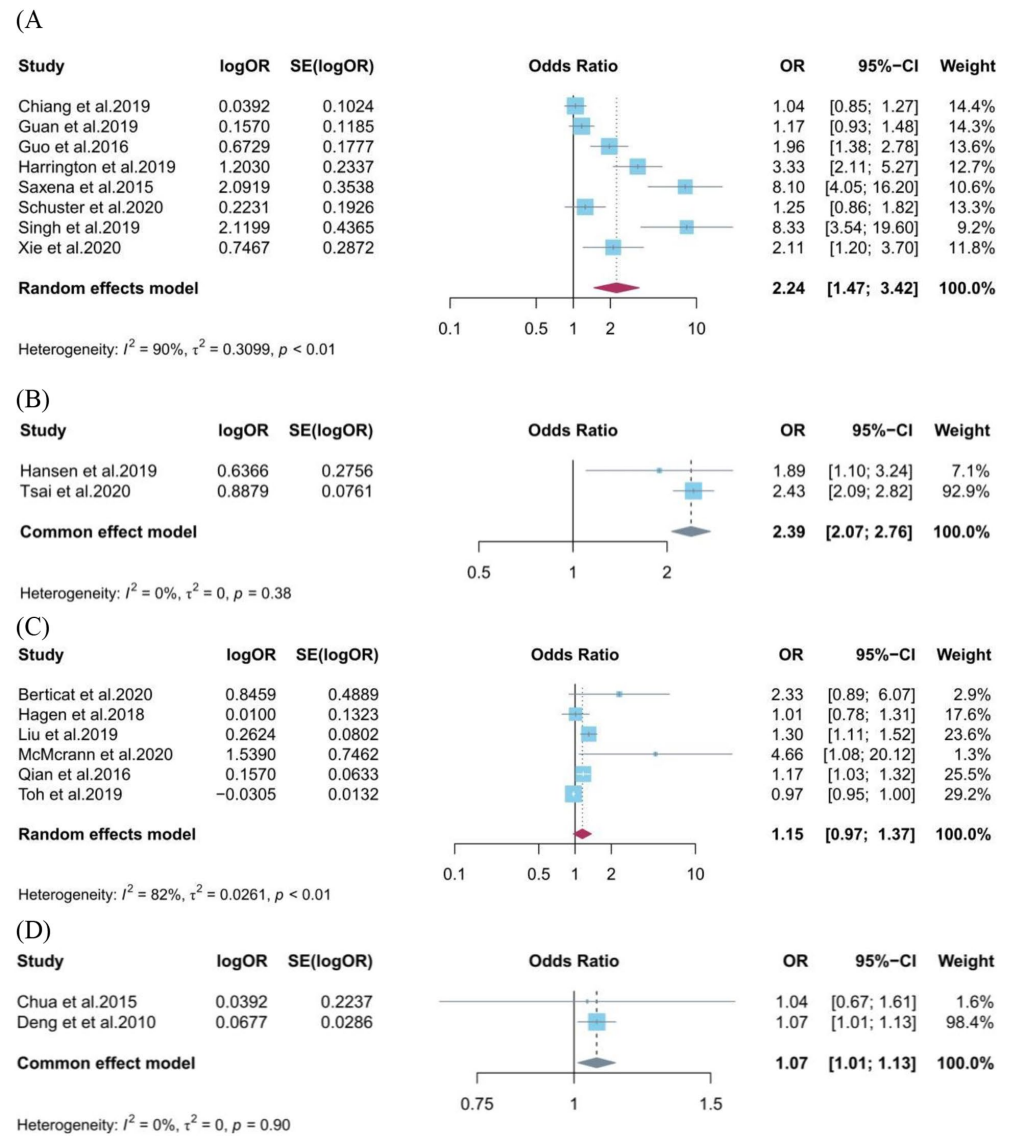
Forest plot for the association between screen time exposure and risk of myopia in children and adolescents. (**A**) screen time (high vs. low) and myopia in cross-sectional studies; (**B**) screen time (high vs. low) and myopia in cohort studies; (**C**) screen time (per 1 h/d increase) and myopia in cross-sectional studies; (**D**) screen time (per 1 h/d increase) and myopia in cohort studies

A total of 11 studies involving 90,415 participants were related to the relationship between categorical exposure to screen time (high vs. low) and myopia in children and adolescents [25–28, 43–46, 49, 50, 53]. We found a significantly higher odds ratio of myopia in the highest category of screen time exposure in cross-sectional studies (OR = 2.24, 95%CI: 1.47–3.42), compared to the lowest category (Fig. 2A). A significant relationship between categorical exposure to screen time and myopia was also observed in cohort studies (OR = 2.39, 95%CI: 2.07–2.79) (Fig. 2B). In the subgroup analysis stratified by screen device type, we found screen time exposure (high vs. low) from computers (OR = 8.19, 95%CI: 4.78–14.04) and televisions (OR = 1.46, 95%CI: 1.02–2.10) were significantly related to myopia in cross-sectional studies, while not from smartphones (OR = 1.94, 95%CI: 0.70–5.39) (Figure S1). According to the subgroup analysis stratified by quality assessment, in cross-sectional studies, we observed significant associations in the high quality group (OR = 2.94, 95%CI: 1.50–5.76), while the moderate quality group not (OR = 1.59, 95%CI: 0.85–2.97) (Figure S2). In the subgroup analysis stratified by geographic region, we found significant associations in East Asia (OR = 1.62, 95%CI: 1.07–2.45), and South Asia (OR = 8.19, 95%CI: 4.78–14.04), while not in Europe and America (OR = 1.59, 95%CI: 0.85–2.97) (Figure S3). In addition, we performed a subgroup analysis stratified by research period, and observed significant associations in the research period after 2008 group (OR = 2.99, 95%CI: 1.67–5.35), while the research period before 2008 group not (OR = 1.08, 95%CI: 0.91–1.29). Due to the limited number of studies included, we did not perform subgroup analysis in cohort studies.

A total of 8 studies involving 11,925 participants were included for the association between continuous exposure (per 1 h/d increase) to screen time and myopia in children and adolescents [29, 30, 47, 48, 51, 52, 54, 55]. We found no association between screen time exposure with 1-hour increase per day and myopia in cross-sectional studies (OR = 1.15, 95%CI: 0.97–1.37) (Fig. 2C), however, a significant association was observed in cohort studies (OR = 1.07, 95%CI: 1.01–1.13) (Fig. 2D). According to the subgroup analysis stratified by screen device type, we observed significant associations between screen time exposure (per 1 h/d increase) from computers (OR = 1.22, 95%CI: 1.10–1.35) was associated with myopia in cross-sectional studies, while not from smartphones (OR = 1.78, 95%CI: 0.40–7.97) (Figure S5). We did not perform subgroup analysis stratified by quality assessment and research period for continuous exposure to screen time and myopia, as all relevant studies were considered high quality and their research period was after 2008. Furthermore, subgroup analysis stratified by geographic region showed significant associations in East Asia (OR = 1.22, 95%CI: 1.10–1.35), while not in Europe and America (OR = 1.15, 95%CI: 0.75–4.37) (Figure S6).

### Publication bias and sensitivity analysis

**Fig. 3:**
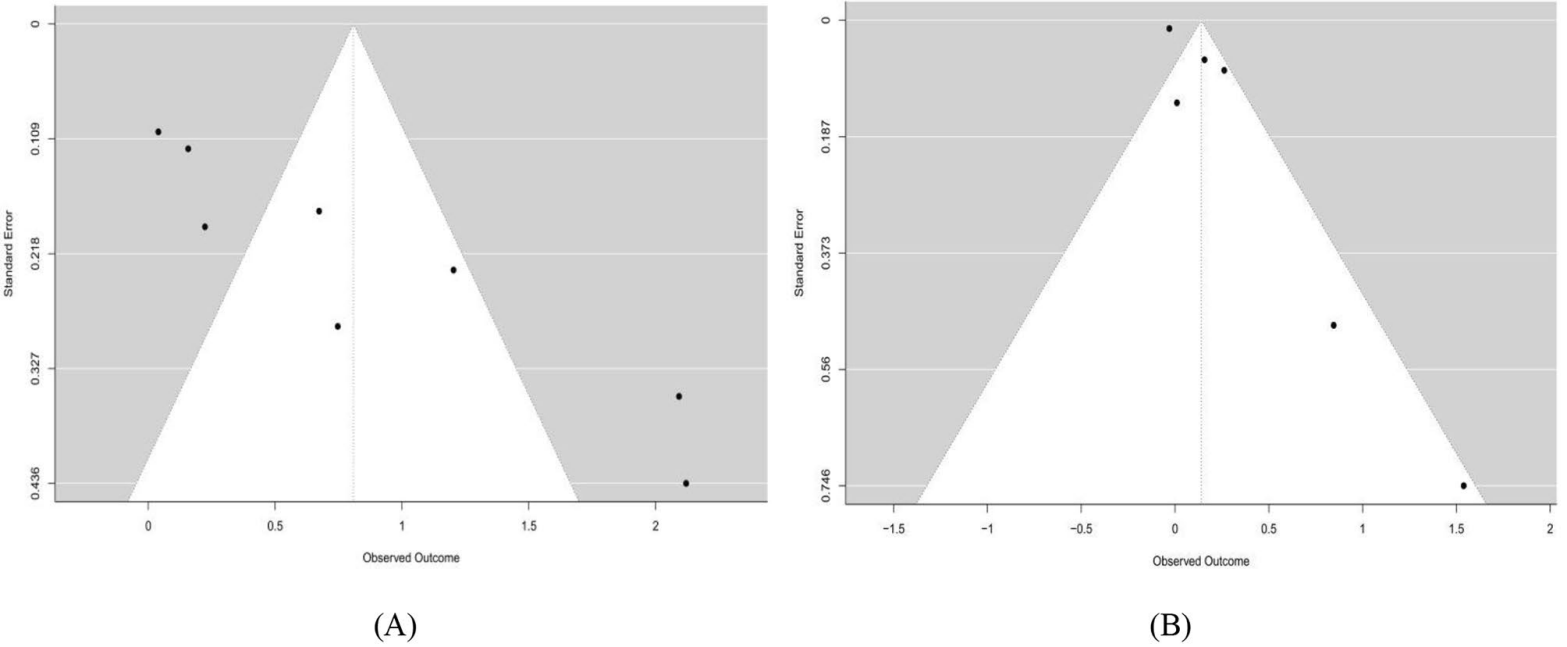
Funnel plots show the risk of publication bias in the meta-analysis. (**A**) screen time (high vs. low) and myopia in cross-sectional studies; (**B**) screen time (per 1 h/d increase) and myopia in cross-sectional studies

**Fig. 4:**
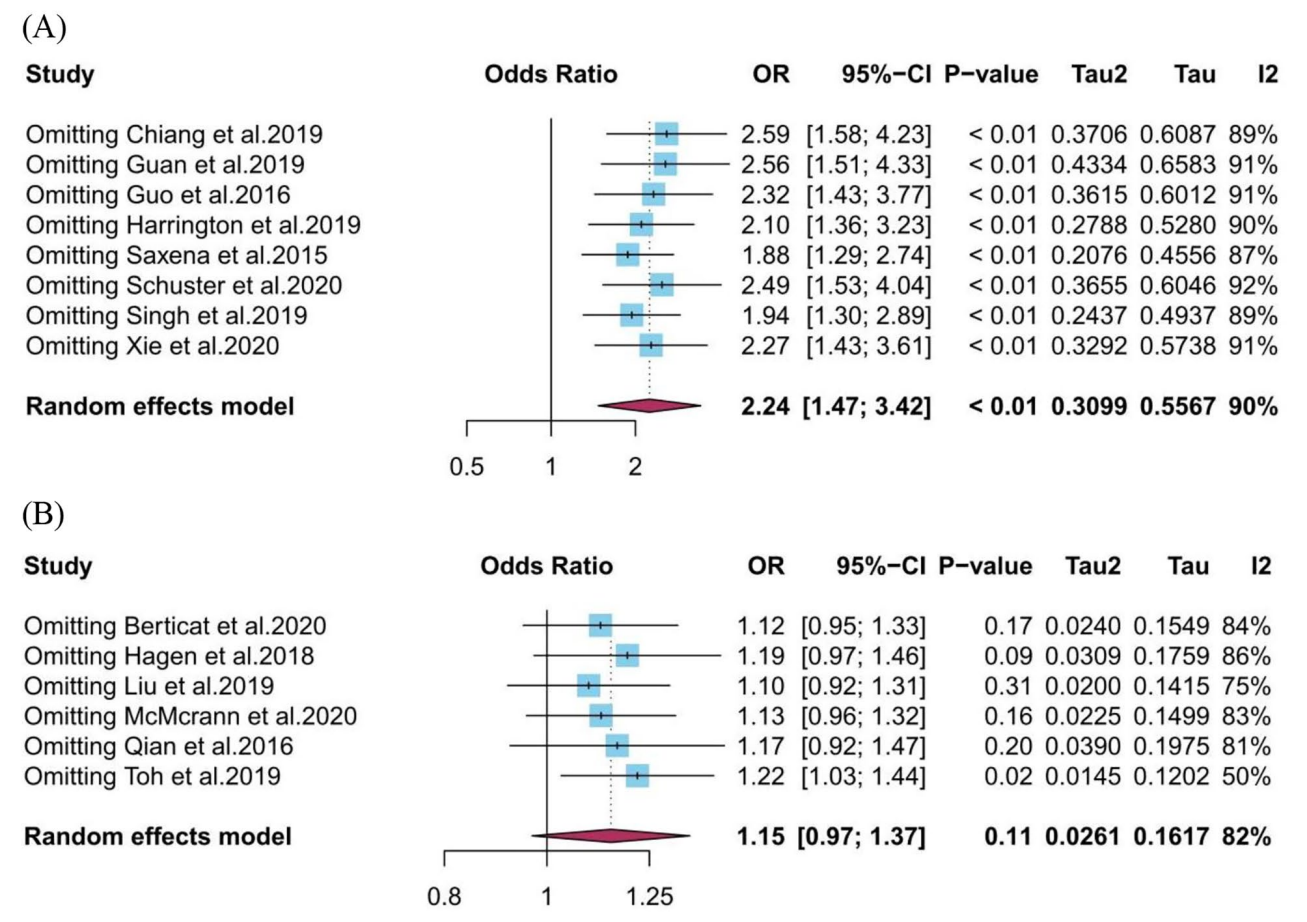
Sensitivity analysis using leave-one-out method for the association between screen time exposure and myopia. (**A**) screen time (high vs. low) and myopia in cross-sectional studies; (**B**) screen time (per 1 h/d increase) and myopia in cross-sectional studies

Funnel plots and Egger’s test were performed to estimate publication bias in cross-sectional studies. Due to the limited number of studies included, we were unable to perform publication bias detection and sensitivity analysis for cohort studies. In cross-sectional studies, publication bias was detected in the screen time (high vs. low)-myopia group (Fig. 3A). The *P* value of Egger’s test in this group was 0.012, indicating the existence of publication bias. After trim and fill analysis, the pooled OR (95%CI) was 1.34 (1.18–1.52) in this exposure-outcome group. A similar situation was also found in screen time (per 1 h/d increase) and myopia (Fig. 3B). The *P* value of Egger’s test in this group was 0.028, indicating the existence of publication bias. After trim and fill analysis, the pooled OR (95%CI) was 1.02 (0.86–1.20). Our sensitivity analysis using the leave-one-out method presented that the combined results are generally robust in the screen time (high vs. low)-myopia group (Fig. 4A), while not in the screen time (per 1 h/d increase)-myopia group (Fig. 4B).

## Discussion

In recent years, the influence of screen time exposure on children’s vision has attracted worldwide attention. Our study provided significant evidence for the correlation between screen time exposure and myopia. To our knowledge, this study is the largest and most comprehensive systematic review and meta-analysis of screen time exposure and myopia in children and adolescents. Meanwhile, this study provided evidence for the association between screen time exposure from different devices and myopia. Specifically, we acknowledge that the degree of association between the use of certain screen devices and myopia is relatively small. However, it should be pointed out that considering the widespread exposure to screen time, the combined effects at the population level may translate into a noticeable rise in the number of myopic individuals.

In this study, we conducted a systematic review to examine the association between screen time exposure and myopia in children and adolescents. The findings of this meta-analysis showed that categorical exposure and continuous exposure to screen time were both related to myopia. What’s more, we discovered that screen time exposures from computers and televisions were significantly associated with myopia, while smartphones were not. This discovery may indicate that computer screens were more capable of generating myopic signals than other screen devices. Of course, the heterogeneity contained in these analytical models deserve a careful explanation of the results. According to the view from Swiatczak and Schaeffel, smartphones may not be likely to cause myopia because their letter sizes minimize center-surround effects, which may be important in linking reading to myopia development [56]. Currently, due to the digitalization of education, controlling computer screen time may be more challenging than controlling the use of digital smart devices, which are often used more for leisure [31].

Our findings differ from previous systematic reviews in several aspects. For example, a meta-analysis carried out by Foreman et al. (2021) included 11 observational studies (including five prospective cohort studies), and found that the use of smart devices may be related to myopia prevalence [31]. However, Lanca et al. (2020) reviewed five observational studies (including two prospective cohort studies), and reported that there was no statistical significance between screen time exposure and myopia (pooled OR = 1.02, 95 CI% 0.96–1.08) [57]. Foreman et al. believed that the reason for this result was that some insignificant ORs in their models came from significant OR transformations in the source article, which may have led to the absence of associations observed in Lanca’s meta-analysis [31]. At the same time, both meta-analyses combined continuous and categorical data of screen time exposure together without separating them, which may lead to deviations in statistical results. In addition, Foreman et al. and Lanca et al. did not include television viewing as part of screen time, and key studies on the relationship between television screen time and myopia included in our study were excluded from their meta-analysis. Of course, it is permissible for these systematic reviews to make inconsistent conclusions considering the differences in test methods for screen time and the confounder of near work. Furthermore, it should be highlighted that the majority of the research included in this analysis was published within the last five years, making this study timely, comprehensive, and significant for public health.

Most of the studies included in this study confirmed myopia cases through cycloplegic refraction. However, several studies have obtained relevant data in the form of self-reported myopia through questionnaires [28, 51, 52, 55]. Self-reported myopia assessments may be used for two different aspects. Firstly, self-reported measurements enable quick surveys and extensive population coverage. For example, this is particularly critical during the COVID-19 epidemic, as face-to-face expert ophthalmic examinations are not feasible considering safe distance measures and the closure of optometry clinics [55]. Secondly, a previous study by Cumberland et al. showed no statistically significant difference between detailed ophthalmic assessment and self-reported myopia, and suggested using self-reported outcomes as a reasonable and accurate alternative method to confirm refractive status in a large sample size population-based questionnaire [58].

It is necessary to incorporate the analysis of the relationship between screen usage and myopia in historical and social contexts. Our subgroup analysis revealed that screen time exposure was associated with myopia in East Asia and South Asia, while in Europe and America not. Meanwhile, we also observed that screen time was associated with myopia after 2008. The epidemic of myopia was well-established in most of those Asia regions, such as Taiwan, Hongkong, Singapore, and Chinese mainland, before screen devices were in large-scale use [59–62]. It is important to recognize that the Internet did not become publicly accessible until 1993, and digital smart devices did not emerge until 2008 [39]. This has significant implications for myopia prevention strategies. If traditional book reading could lead to a widespread increase in myopia, then a prevention approach focused on reducing screen time in favor of book reading might not be effective. Global education authorities tend to focus on screen time as an issue, probably because it means they can avoid solving the more complex problems around total near-work and outdoor time. Similar to reading and studying (e.g., doing homework, and writing), using computers, playing video games, watching TV, and using smartphones/tablets are also considered near-work activities [7, 31]. More time spent on near-work activities was associated with higher odds of myopia [7]. This implies that a more crucial objective should be to limit the duration and frequency of near-work activities, regardless of the medium used. According to Morgan et al.‘s opinion, if future studies confirm a definite causal relationship between screen time and myopia, there is a chance that restricting screen device usage alone could result in children returning to previous habits that contributed to myopia, such as long-time near work associated with reading books and writing [39]. Without supplementary measures, limiting digital device usage may not significantly affect myopia rates. Furthermore, screen usage could also be harmful if it diverts children from spending time outdoors [25]. Simply limiting screen time may have little effect on preventing myopia in children and adolescents. Active promotion of outdoor activity during daylight may help delay the onset and slow myopia progression in childhood and adolescence [63, 64]. Previous epidemiological studies have provided longitudinal evidence and cross-sectional evidence of a protective effect of outdoor time [65, 66]. Rose et al. highlighted the potential for preventive measures, suggesting a well-defined biological pathway that could mediate a causal effect. Specifically, they proposed that exposure to brighter outdoor light could enhance dopamine release, subsequently inhibiting axial elongation of the eyeball [66]. This proposition is supported by compelling evidence from animal studies, which demonstrate that increased ambient light exposure can trigger these beneficial neurochemical and anatomical changes [67, 68]. The increased time outdoors has become the leading approach to the prevention of the onset of myopia. If the established pattern of spending more time indoors persists, it is necessary to take active steps to encourage children to go outdoors.

Myopia is rare among children with limited or no access to schooling. However, as education systems advance and more children receive formal education, the incidence of myopia after 12 years of schooling rises to approximately 20% [39]. In the school environment, there’s a tendency for children with longer durations of education to exhibit higher rates of myopia. Moreover, among students at the same educational level, those who excel academically and pursue more rigorous academic paths tend to have a higher prevalence of myopia [59]. Mendelian randomization analyses have indicated the influence of educational attainment on myopia [69–71]. The increase in myopia prevalence throughout school education is mainly due to the increase in environmental risk factors encountered during the school year, rather than simply from advancing age [72]. The epidemiological link between additional tutoring and the development of myopia has also been studied [59]. Especially in South Korea, it is now estimated that more than 75% of schoolchildren receive some private tutoring, for nearly 7 h a week [73]. In 2006, the cost of additional tutoring for parents in South Korea amounted to 2.57% of the country’s gross domestic product (GDP), surpassing the government’s education budget and representing nearly 20% of household income [74]. By 2016, South Korea boasted over 70,000 registered private tutoring institutions, with a majority located in Seoul [75]. This could potentially elucidate the 96.5% prevalence of myopia among young males in Seoul [76]. With the development of technology and GDP, educational pressure may lead students to use screen devices such as smartphones and computers for long periods of time to learn online courses, receive additional tutoring or practice, and increase the incidence of myopia.

The main advantage of this study is that it provides the latest and most comprehensive evidence on the association between screen time exposure and myopia in children and adolescents. Second, we pooled effect estimates from categorical and continuous exposure to screen time separately. Previous meta-analyses in related fields did not consider to separate processing of categorical data and continuous data, and this would compromise the accuracy of pooled results [31, 57]. Third, this meta-analysis extracted data from multivariate-adjusted models, which to some extent reduced the influence of confounding factors. Further, compared to previous meta-analyses, we conducted an in-depth analysis for screen device type, study quality geographic region, and research period to reveal potential patterns and influencing factors, thereby providing more details and insights for understanding this field.

Despite these advantages, some limitations should also be mentioned. First, the majority of the studies included in this study are cross-sectional studies, and the associations reported in these studies cannot prove causal relationships. Second, we found high heterogeneity in included cross-sectional studies, which may be due to differences in research design, population characteristics, exposure levels, and outcome evaluation in included literature, or perhaps heterogeneity comes from trying to add activities that really are not functioning in the same way, making a cumulative estimate inappropriate; due to the small number of included cohort studies and over 90% of the weight coming from one study, this may result in statistical analysis not being able to have accurate and reliable pooled effect estimates and heterogeneity. Third, all included studies are divergent in their measurement methods and geographic regions, the results of self-reported myopia or lack of ciliary muscle paralysis may not be as accurate as those of studies performing ciliary muscle paralysis; research in developing countries provides different myopia prevalence compared to developed countries, however, a meta-analysis works best when studies are expected to give broadly similar results. Unavoidable differences in the included studies can affect the effectiveness of meta-analysis in this study. Fourth, the studies included in this study were all based on data obtained from self-reported questionnaires for screen time. Considering that participants tend to underestimate their screen time, the study may be influenced by recall bias [77]. Fifth, adequate outdoor time has been proven to be a protective factor for myopia. However, some of the literature we included in the meta-analysis did not adjust for important confounding factors such as outdoor activities or outdoor time, which may interfere with the final results [8]. Finally, because most of the literature included did not provide effect values for the association between screen distance and myopia, we are unable to further explore the impact of usage distance from screen devices such as computers, televisions, and smartphones on myopia.

The results of this study support the hypothesis that screen time exposure may be related to myopia in children and adolescents, but the direction and degree of these associations may vary depending on the type of screen device and individual screen time. More epidemiological research, particularly prospective cohort studies, is needed to confirm these potential associations, nevertheless. Here are some ideas for resolving the remaining open issues in this area:

- In addition to screen time exposure from computers and televisions, the independent impact of smartphones and tablets on myopia has not been fully explored, and there is a lack of research in related fields. And compared to other screen devices, smartphones and tablets have closer viewing distances, so further independent evaluation should be conducted in future research.
- At present, almost all screen time data is obtained through self-reported questionnaires. In order to reduce the impact of recall bias, a feasible method is to install an application on the screen device that can track real-time usage, allowing accurate investigation of the dose-dependent impact of screen time on myopia in longitudinal studies [31].
- In included studies, cross-sectional methods are still widely used to describe screen time exposure and myopia, and further cohort studies are needed to clarify its potential causal relationship.
- More systematic investigations on categorical exposure and continuous exposure to screen time are needed.

### Electronic supplementary material

Below is the link to the electronic supplementary material.

Supplementary Material 1

## Data Availability

Upon reasonable request, the corresponding author (Shaojun Xu) will provide the dataset generated and/or analyzed in the current study (Email: xushaojun@ahmu.edu.cn).
