# Supplementary material for: The association between screen time exposure and myopia in children and adolescents: a meta-analysis
Source: BMC Public Health. 2024 Jun 18;24:1625. doi: 10.1186/s12889-024-19113-5 (PMC11186094; doi:10.1186/s12889-024-19113-5)
Supplement: Supplementary file 1 — Supplementary Material 1 [file 12889_2024_19113_MOESM1_ESM.docx]

**Supplementary Material**

**The Association Between Screen Time Exposure and Myopia in Children and Adolescents: A Meta-Analysis**

Zhiqiang Zong^1^, Yaxin Zhang^2^, Jianchao Qiao^1^, Yuan Tian^3^, Shaojun Xu^2,4,1^

^1.^ The Second School of Clinical Medicine, Anhui Medical University, 81 Meishan Road, Hefei, 230032, Anhui, China

^2^ Department of Maternal, Child and Adolescent Health, School of Public Health, Anhui Medical University, 81 Meishan Road, Hefei 230032, Anhui, China

^3^ The First School of Clinical Medicine, Anhui Medical University, 81 Meishan Road, Hefei, 230032, Anhui, China

^4^ MOE Key Laboratory of Population Health Across Life Cycle, 81 Meishan Road, Hefei 230032, Anhui, China

**Corresponding author:** Shao-Jun Xu, Email: [xushaojun@ahmu.edu.cn](mailto:xushaojun@ahmu.edu.cn)

**Table of contents**

| **Table S1.** Preferred Reporting Items for Systematic Reviews and Meta-Analysis (PRISMA) 2020 Checklist. |
| --- |
| **Table S2.** Database search term list. |
| **Table S3.** Newcastle-Ottawa Quality Assessment Scale for cohort studies. |
| **Table S4.** Newcastle-Ottawa Quality Assessment Scale for cross-sectional studies. |
| **Table S5.** Reasons for the exclusion of 20 articles in the full-text rescreening. |
| **Table S6.** Evaluation of risk of bias using the Newcastle Ottawa Scale (NOS) for 18 observational studies included in the meta-analysis |
| **Figure S1.** Subgroup analysis stratified by screen device type in cross-sectional studies, forest plots for the association between categorical exposure to screen time (high vs. low) and myopia in children and adolescents. |
| **Figure S2.** Subgroup analysis stratified by study quality in cross-sectional studies, forest plots for the association between categorical exposure to screen time (high vs. low) and myopia in children and adolescents. |
| **Figure S3.** Subgroup analysis stratified by geographic region in cross-sectional studies, forest plots for the association between categorical exposure to screen time (high vs. low) and myopia in children and adolescents. |
| **Figure S4.** Subgroup analysis stratified by research period in cross-sectional studies, forest plots for the association between categorical exposure to screen time (high vs. low) and myopia in children and adolesce |
| **Figure S5.** Subgroup analysis stratified by screen device type in cross-sectional studies, forest plots for the association between continuous exposure to screen time (per 1h/d increase) and myopia in children and adolescents. |
| **Figure S6.** Subgroup analysis stratified by geographic region in cross-sectional studies, forest plots for the association between continuous exposure to screen time (per/d 1h increase) and myopia in children and adolescents. |

**Table S1.** Preferred Reporting Items for Systematic Reviews and Meta-Analysis (PRISMA) 2020 Checklist.

| **Section and Topic** | **Item #** | **Checklist item** | **Location where item is reported** |
| --- | --- | --- | --- |
| **TITLE** | | |  |
| Title | 1 | Identify the report as a systematic review. | Page 1 |
| **ABSTRACT** | | |  |
| Abstract | 2 | See the PRISMA 2020 for Abstracts checklist. | Page 1-2 |
| **INTRODUCTION** | | |  |
| Rationale | 3 | Describe the rationale for the review in the context of existing knowledge. | Page 2-3 |
| Objectives | 4 | Provide an explicit statement of the objective(s) or question(s) the review addresses. | Page 2-3 |
| **METHODS** | | |  |
| Eligibility criteria | 5 | Specify the inclusion and exclusion criteria for the review and how studies were grouped for the syntheses. | Page 3-4 |
| Information sources | 6 | Specify all databases, registers, websites, organizations, reference lists and other sources searched or consulted to identify studies. Specify the date when each source was last searched or consulted. | Page 3-4 |
| Search strategy | 7 | Present the full search strategies for all databases, registers and websites, including any filters and limits used. | Page 3-4,Supplemental Material Table S2 |
| Selection process | 8 | Specify the methods used to decide whether a study met the inclusion criteria of the review, including how many reviewers screened each record and each report retrieved, whether they worked independently, and if applicable, details of automation tools used in the process. | Page 4， Supplemental Material Table S5 |
| Data collection process | 9 | Specify the methods used to collect data from reports, including how many reviewers collected data from each report, whether they worked independently, any processes for obtaining or confirming data from study investigators, and if applicable, details of automation tools used in the process. | Page 4 |
| Data items | 10a | List and define all outcomes for which data were sought. Specify whether all results that were compatible with each outcome domain in each study were sought (e.g. for all measures, time points, analyses), and if not, the methods used to decide which results to collect. | Page 4 |
|  | 10b | List and define all other variables for which data were sought (e.g. participant and intervention characteristics, funding sources). Describe any assumptions made about any missing or unclear information. | Page 4 |
| Study risk of bias assessment | 11 | Specify the methods used to assess risk of bias in the included studies, including details of the tool(s) used, how many reviewers assessed each study and whether they worked independently, and if applicable, details of automation tools used in the process. | Page 4，Supplemental Material Table S3-4 |
| Effect measures | 12 | Specify for each outcome the effect measure(s) (e.g. risk ratio, mean difference) used in the synthesis or presentation of results. | Page 4 |
| Synthesis methods | 13a | Describe the processes used to decide which studies were eligible for each synthesis (e.g. tabulating the study intervention characteristics and comparing against the planned groups for each synthesis (item #5)). | Page 4-5 |
|  | 13b | Describe any methods required to prepare the data for presentation or synthesis, such as handling of missing summary statistics, or data conversions. | Page 5 |
|  | 13c | Describe any methods used to tabulate or visually display results of individual studies and syntheses. | Page 5 |
|  | 13d | Describe any methods used to synthesize results and provide a rationale for the choice(s). If meta-analysis was performed, describe the model(s), method(s) to identify the presence and extent of statistical heterogeneity, and software package(s) used. | Page 5 |
|  | 13e | Describe any methods used to explore possible causes of heterogeneity among study results (e.g. subgroup analysis, meta-regression). | Page 5 |
|  | 13f | Describe any sensitivity analyses conducted to assess robustness of the synthesized results. | Page 5 |
| Reporting bias assessment | 14 | Describe any methods used to assess risk of bias due to missing results in a synthesis (arising from reporting biases). | Page 5 |
| Certainty assessment | 15 | Describe any methods used to assess certainty (or confidence) in the body of evidence for an outcome. | NA |
| **RESULTS** | | |  |
| Study selection | 16a | Describe the results of the search and selection process, from the number of records identified in the search to the number of studies included in the review, ideally using a flow diagram. | Page 5, Figure 1 |
|  | 16b | Cite studies that might appear to meet the inclusion criteria, but which were excluded, and explain why they were excluded. | Page 5 |
| Study characteristics | 17 | Cite each included study and present its characteristics. | Page 5, Table 1 |
| Risk of bias in studies | 18 | Present assessments of risk of bias for each included study. | Page 5-6, Table 2 |
| Results of individual studies | 19 | For all outcomes, present, for each study: (a) summary statistics for each group (where appropriate) and (b) an effect estimate and its precision (e.g. confidence/credible interval), ideally using structured tables or plots. | Page 5-6, Table 1 |
| Results of syntheses | 20a | For each synthesis, briefly summarise the characteristics and risk of bias among contributing studies. | Page 6 |
|  | 20b | Present results of all statistical syntheses conducted. If meta-analysis was done, present for each the summary estimate and its precision (e.g. confidence/credible interval) and measures of statistical heterogeneity. If comparing groups, describe the direction of the effect. | Page 6, Figure 2, Supplemental Material Figure S1-S5 |
|  | 20c | Present results of all investigations of possible causes of heterogeneity among study results. | Page 6 |
|  | 20d | Present results of all sensitivity analyses conducted to assess the robustness of the synthesized results. | Page 6, Figure 4 |
| Reporting biases | 21 | Present assessments of risk of bias due to missing results (arising from reporting biases) for each synthesis assessed. | Page 6, Figure 3 |
| Certainty of evidence | 22 | Present assessments of certainty (or confidence) in the body of evidence for each outcome assessed. | NA |
| **DISCUSSION** | | |  |
| Discussion | 23a | Provide a general interpretation of the results in the context of other evidence. | Page 6-8 |
|  | 23b | Discuss any limitations of the evidence included in the review. | Page 8-9 |
|  | 23c | Discuss any limitations of the review processes used. | Page 8-9 |
|  | 23d | Discuss implications of the results for practice, policy, and future research. | Page 9 |
| **OTHER INFORMATION** | | |  |
| Registration and protocol | 24a | Provide registration information for the review, including register name and registration number, or state that the review was not registered. | NA |
|  | 24b | Indicate where the review protocol can be accessed, or state that a protocol was not prepared. | Not prepared |
|  | 24c | Describe and explain any amendments to information provided at registration or in the protocol. | NA |
| Support | 25 | Describe sources of financial or non-financial support for the review, and the role of the funders or sponsors in the review. | Page 9-11 |
| Competing interests | 26 | Declare any competing interests of review authors. | Page 9-11 |
| Availability of data, code and other materials | 27 | Report which of the following are publicly available and where they can be found: template data collection forms; data extracted from included studies; data used for all analyses; analytic code; any other materials used in the review. | Page 9-11 |

**Table S2.** Database search term list.

| Database | Search term |
| --- | --- |
| PubMed | ("screen time" [Mesh] OR "digital screen"[tiab] OR "electronic screen"[tiab] OR computer[tiab] OR "personal computer"[tiab] OR video[tiab] OR "video game"[tiab] OR "video console"[tiab] OR "computer game"[tiab] OR "digital game"[tiab] OR "electronic game"[tiab] OR television[tiab] OR TV[tiab] OR "watching TV"[tiab] OR "watching television"[tiab] OR "television watching"[tiab] OR tablet[tiab] OR laptop[tiab] OR desktop[tiab] OR smartphone[tiab] OR phone[tiab] OR "cell phone"[tiab] OR "mobile device"[tiab] OR "electronic device"[tiab] OR "handheld device"[tiab] OR Internet[tiab] OR ipad[tiab] OR i-pad[tiab] OR i-phone[tiab] OR iphone[tiab]) AND (myopia[Mesh] OR "refractive error"[tiab] OR nearsightedness[tiab] OR shortsightedness[tiab] OR "poor vision"[tiab] OR "myopic vision"[tiab] OR "blurred vision" [tiab] OR "occular myopia"[tiab]) AND (child [Mesh] OR adolescent[Mesh] OR children [tiab] OR "young children"[tiab] OR kid[tiab] OR "young kid"[tiab] OR teen[tiab] OR adolescence[tiab] OR "young people" [tiab] OR "young person"[tiab] OR youth[tiab] OR juvenile[tiab] OR youngers[tiab] OR newborn[tiab] OR infant[tiab] OR kindergarden [tiab] OR "primary school"[tiab] OR "high school"[tiab] OR "middle school"[tiab] OR boy[tiab] OR girl[tiab]) |
| Embase | 1. ('screen time'/exp OR 'digital screen' OR 'electronic screen' OR computer/exp OR 'personal computer' OR video OR 'video game' OR 'video console' OR 'computer game' OR 'digital game' OR 'electronic game' OR television OR TV OR 'watching TV' OR 'watching television' OR 'television watching'/exp OR 'television watching' OR tablet OR laptop OR desktop OR smartphone OR phone OR 'cell phone' OR 'mobile device' OR 'electronic device'/exp OR 'electronic device' OR 'handheld device' OR Internet OR ipad OR i-pad OR i-phone OR iphone). ti.ab.2. (myopia/exp OR 'refractive error'/exp OR 'refractive error' OR nearsightedness OR 'shortsightedness'/exp OR shortsightedness OR 'poor vision' OR 'myopic vision' OR 'blurred vision'/exp OR 'blurred vision' OR 'occular myopia').ti.ab. 3. (child/exp OR 'young children' OR kid OR 'young kid' OR teenager OR teen OR teenage OR adolescent OR adolescence OR 'young people' OR 'young person' OR youth OR juvenile OR youngers OR infant OR newborn OR kindergarden OR 'primary school' OR 'high school' OR 'middle school' OR boy OR girl).ti.ab.1 AND 2 AND 3 |
| Web of Science | **TS =** ("screen time" OR screen OR "digital screen" OR "electronic screen" OR computer OR "personal computer" OR "computer use" OR video OR "video game" OR "video console" OR "computer game" OR "digital game" OR "electronic game" OR television OR TV OR "watching TV" OR "watching television" OR "television watching" OR tablet OR laptop OR desktop OR smartphone OR phone OR "cell phone" OR "mobile device" OR "electronic device" OR "handheld device" OR Internet OR ipad OR i-pad OR i-phone OR iphone) AND **TS =** (myopia OR "refractive error" OR nearsightedness OR shortsightedness OR "poor vision" OR "myopic vision" OR "blurred vision" OR "occular myopia") AND **TS =** (child OR children OR "young children" OR kid OR "young kid" OR teenager OR teen OR teenage OR adolescent OR adolescence OR "young people" OR "young person" OR youth OR juvenile OR youngers OR infant OR newborn OR "kindergarden" OR "primary school" OR "high school" OR "middle school" OR boy OR girl) Indexes=SCI-EXPANDED, SSCI, CPCI-S, CPCI-SSH Timespan = All years |

**Table S3.** Newcastle-Ottawa Quality Assessment Scale for cohort studies.

| **Selection:** (Maximum 4 stars)  1) Selection of the non exposed cohort:  a) truly representative of the average children and adolescents who are less likely to use screen devices (describe) in the community.  b) somewhat representative of the average children and adolescents who are less likely to use screen devices (describe) in the community.  c) selected group of users eg nurses, volunteers.  d) no description of the derivation of the cohort.  2) Selection of the non exposed cohort:  a) drawn from the same community as the exposed cohort.  b) drawn from a different source.  c) no description of the derivation of the non exposed cohort.  3) Ascertainment of exposure:  a) secure record (eg. Surgical records).  b) structured interview.  c) written self repot.  d) no description.  4) Demonstration that outcome of interest was not present at start of study:  a) yes.  b) no. |
| --- |
| **Comparability:** (Maximum 2 stars)  1) Comparability of cohorts on the basis of the design or analysis:  a) The study controls for the most important factor (age, and gender).  b) The study control for any additional factor (outdoor activity/outdoor time). |
| **Outcome:** (Maximum 4 stars)  1) Assessment of the outcome:  a) Independent blind assessment.  b) Record linkage.  c) Self report.  d) No description.  2) Was follow-up long enough for outcomes to occur:  a) yes (select an adequate follow up period for outcome of interest).  b) no.  Adequacy of follow up of cohorts:  a) complete follow up - all subjects account for.  b) subjects lost to follow up unlikely to introduce bias - small number lost - > 90 % (select an adequate %) follow up, or description provided of those lost).  c) follow-up rate < 90 % (select an adequate %) and no description of those lost.  d) no statement |

**Table S4.** Newcastle-Ottawa Quality Assessment Scale adapted for cross-sectional studies. This scale has been adapted from the Newcastle-Ottawa Quality Assessment Scale for cohort studies to perform a quality assessment of cross-sectional studies for the systematic review.

| **Selection:** (Maximum 5 stars)  1) Representativeness of the sample:  a) Truly representative of the average in the target population. (all subjects or random sampling)  b) Somewhat representative of the average in the target population. (non-random sampling)  c) Selected group of users.  d) No description of the sampling strategy.  2) Sample size:  a) Justified and satisfactory (> 1000).  b) Not justified.  3) Non-respondents:  a) Comparability between respondents and non-respondents characteristics is established, and the response rate is satisfactory.  b) The response rate is unsatisfactory, or the comparability between respondents and non-respondents is unsatisfactory.  c) No description of the response rate or the characteristics of the responders and the non-responders.  4) Ascertainment of the exposure (risk factor):  a) Validated measurement tool.  b) Non-validated measurement tool, but the tool is available or described.  c) No description of the measurement tool. |
| --- |
| **Comparability:** (Maximum 2 stars)  1) The subjects in different outcome groups are comparable, based on the study design or analysis. Confounding factors are controlled.  a) The study controls for the most important factor (age, and gender).  b) The study control for any additional factor (outdoor activity/outdoor time). |
| **Outcome:** (Maximum 3 stars)  1) Assessment of the outcome:  a) Independent blind assessment.  b) Record linkage.  c) Self report.  d) No description.  2) Statistical test:  a) The statistical test used to analyze the data is clearly described and appropriate, and the measurement of the association is presented, including confidence intervals and the probability level (p value).  b) The statistical test is not appropriate, not described or incomplete. |

**Table S5.** Reasons for the exclusion of 20 articles in the full-text rescreening.

| No. | Studies excluded | PMID | Reasons for exclusion of studies |
| --- | --- | --- | --- |
| 1 | Alvarez-Peregrina et al., 2019 | 31531234 | This article did not provide a valuable effect value. Multivariate logistic regression was not used to analyze the probability of being myopic, and estimating ORs (95CIs) were not presented. |
| 2 | Foreman et al., 2021 | 34625399 | This article is a systematic review and meta-analysis. |
| 3 | Hsu et al., 2016 | 27349942 | This article did not provide a clear screen time, and the classification only includes yes or no/unknown. |
| 4 | Hsu et al., 2017 | 28315834 | This article did not provide a clear screen time, and the classification only includes yes or no/unknown. |
| 5 | Huang et al., 2019 | 30855486 | This article did not adjust for confounding factors and not conduct multivariate logistic regression analysis. |
| 6 | Jones et al., 2007 | 17652719 | This article did not adjust for confounding factors and not conduct multivariate logistic regression analysis. |
| 7 | Lanca et al., 2020 | 31943280 | This article is a systematic review and meta-analysis. |
| 8 | Liu et al., 2021 | 34113588 | This article reported the myopia progression, not prevalence. |
| 9 | Lu et al., 2009 | 19506196 | This article did not provide a valuable effect value. Multivariate logistic regression was not used to analyze the probability of being myopic, but estimating ORs (CIs) for the association between watching television (per 1h/d increase) and myopia were not presented. |
| 10 | Ma et al., 2022 | 35695548 | This article did not provide a valuable effect value. Multivariate logistic regression was not used to analyze the probability of being myopic, and estimating ORs (CIs) were not presented. |
| 11 | Matsumura et al., 2022 | 35812505 | This article did not provide a valuable effect value. Multivariate logistic regression model was used, but estimating ORs (CIs) were not presented. |
| 12 | Saxena et al., 2017 | 29253002 | This article reported the myopia progression, not prevalence. |
| 13 | Terasaki et al., 2017 | 28697750 | This article did not provide a valuable effect value. Logistic regression model was used, but estimating ORs (95%CIs) were not presented. |
| 14 | Toh et al., 2020 | 32174368 | This article did not adjust for confounding factors and not conduct multivariate logistic regression analysis. |
| 15 | Morgan et al., 2021 | 33909035 | This article was a review for the evidence linking increased screen time to myopia. |
| 16 | Wang et al., 2021 | 33996724 | This article did not provide a valuable effect value. Logistic regression model was used, but estimating ORs (95%CIs) were not presented. |
| 17 | Wang et al., 2021 | 33443542 | This article did not provide a valuable effect value. Multivariate logistic regression was not used to analyses the probability of being myopic, and estimating ORs (95%CIs) were not presented. |
| 18 | Wong et al., 2021 | 32738229 | This article was a review for the impact of increased digital device usage arising during the COVID-19 pandemic on myopia. |
| 19 | Wu et al., 2019 | 20868261 | This article did not provide a clear screen time, and the classification only includes seldom or none/often. |
| 20 | Yang et al.,2020 | 32046062 | This article did not provide a valuable effect value. Multivariate logistic regression was not used to analyses the probability of being myopic, and estimating ORs (95CIs) were not presented. They applied cox regression model and gave prevalence ratio (PR). |

**Table S6.** Evaluation of risk of bias using the Newcastle Ottawa Scale (NOS) for 19 observational studies included in the meta-analysis.

| Reference | Study design | Selection | | | | Comparability | Outcome | | | Total score |
| --- | --- | --- | --- | --- | --- | --- | --- | --- | --- | --- |
|  |  | Representactive-ness of the sample | Sample size | Non-respondents | Ascertainment of exposure | Based on design and analysis | Assessment of outcome |  | Statistical test |  |
| Berticat et al., 2020 | Cross-sectional | ⋆ |  | ⋆ | ⋆ | ⋆⋆ | ⋆⋆ |  | ⋆ | 8/10 |
| Chiang et al., 2019 | Cross-sectional | ⋆ | ⋆ | ⋆ | ⋆ |  | ⋆ |  | ⋆ | 6/10 |
| Hagen et al., 2018 | Cross-sectional | ⋆ |  | ⋆ | ⋆ | ⋆ | ⋆⋆ |  | ⋆ | 7/10 |
| Harrington et al., 2019 | Cross-sectional | ⋆ | ⋆ | ⋆ | ⋆ |  | ⋆⋆ |  | ⋆ | 7/10 |
| Harrington et al., 2023 | Cross-sectional | ⋆ |  | ⋆ | ⋆ |  | ⋆⋆ |  | ⋆ | 6/10 |
| Guan et al., 2019 | Cross-sectional | ⋆ | ⋆ | ⋆ | ⋆ | ⋆⋆ | ⋆⋆ |  | ⋆ | 9/10 |
| Guo et al., 2016 | Cross-sectional | ⋆ | ⋆ | ⋆ | ⋆ | ⋆ | ⋆⋆ |  | ⋆ | 8/10 |
| Liu et al., 2019 | Cross-sectional | ⋆ |  | ⋆ | ⋆ | ⋆⋆ | ⋆⋆ |  | ⋆ | 8/10 |
| McMcrann et al., 2020 | Cross-sectional | ⋆ |  | ⋆ | ⋆ | ⋆ | ⋆ |  | ⋆ | 6/10 |
| Qian et al., 2016 | Cross-sectional | ⋆ | ⋆ | ⋆ | ⋆ | ⋆⋆ | ⋆⋆ |  | ⋆ | 9/10 |
| Saxena et al., 2015 | Cross-sectional | ⋆ | ⋆ | ⋆ | ⋆ | ⋆⋆ | ⋆⋆ |  | ⋆ | 9/10 |
| Schuster et al., 2020 | Cross-sectional | ⋆ | ⋆ | ⋆ | ⋆ |  | ⋆ |  | ⋆ | 6/10 |
| Singh et al., 2019 | Cross-sectional | ⋆ | ⋆ | ⋆ | ⋆ | ⋆⋆ | ⋆⋆ |  | ⋆ | 9/10 |
| Toh et al., 2019 | Cross-sectional | ⋆ | ⋆ | ⋆ | ⋆ | ⋆⋆ | ⋆ |  | ⋆ | 8/10 |
| Xie et al., 2020 | Cross-sectional | ⋆ |  | ⋆ | ⋆ | ⋆⋆ | ⋆ |  | ⋆ | 7/10 |
| Reference | Study design | Selection | | | | Comparability | Exposure | | | Total score |
|  |  | Representactive-ness of the exposed cohort | Selection of the non-exposed cohort | Ascertainment of exposure | Demonstration that outcome of interest was not present at start of the study | Based on design and analysis | Assessment of outcome | Was follow-up long enough for the outcome | Adequacy of follow-up of cohorts |  |
| Chua et al., 2015 | Cohort | ⋆ | ⋆ | ⋆ |  | ⋆ | ⋆⋆ | ⋆ | ⋆ | 8/10 |
| Deng et al., 2010 | Cohort | ⋆ | ⋆ | ⋆ |  |  | ⋆ | ⋆ | ⋆ | 6/10 |
| Tsai et al., 2020 | Cohort | ⋆ | ⋆ | ⋆ |  | ⋆⋆ | ⋆⋆ | ⋆ | ⋆ | 9/10 |
| Hansen et al., 2019 | Cohort | ⋆ | ⋆ | ⋆ |  | ⋆⋆ | ⋆⋆ | ⋆ | ⋆ | 9/10 |

**Figure S1.** Subgroup analysis stratified by screen device type in cross-sectional studies, forest plots for the association between categorical exposure to screen time (high vs. low) and myopia in children and adolescents.

**Figure S2.** Subgroup analysis stratified by study quality in cross-sectional studies, forest plots for the association between categorical exposure to screen time (high vs. low) and myopia in children and adolescents.

**Figure S3.** Subgroup analysis stratified by geographic region in cross-sectional studies, forest plots for the association between categorical exposure to screen time (high vs. low) and myopia in children and adolescents.

**Figure S4.** Subgroup analysis stratified by research period in cross-sectional studies, forest plots for the association between categorical exposure to screen time (high vs. low) and myopia in children and adolescents.

**Figure S5.** Subgroup analysis stratified by screen device type in cross-sectional studies, forest plots for the association between continuous exposure to screen time (per 1h/d increase) and myopia in children and adolescents.

**Figure S6.** Subgroup analysis stratified by geographic region in cross-sectional studies, forest plots for the association between continuous exposure to screen time (per 1h/d increase) and myopia in children and adolescents.
